# Hypobaric hypoxia and cardiac baroreflex sensitivity in young women

**DOI:** 10.1152/ajpheart.00452.2022

**Published:** 2022-10-14

**Authors:** James P. Fisher, Johanna Roche, Rachel Turner, Anna Walzl, Giulia Roveri, Hannes Gatterer, Christoph Siebenmann

**Affiliations:** ^1^Manaaki Manawa–The Centre for Heart Research, Department of Physiology, Faculty of Medical & Health Sciences, University of Auckland, Auckland, New Zealand; ^2^Institute of Mountain Emergency Medicine, Eurac Research, Bolzano, Italy; ^3^Department of Anaesthesiology, LMU Klinikum, Ludwig-Maximilians-University München, Munich, Germany

**Keywords:** altitude, baroreflex, human, hypoxia, women

## Abstract

We sought to determine the effects of prolonged moderate hypobaric hypoxia (HH) on cardiac baroreflex sensitivity (cBRS) in young women and whether these effects are a consequence of the reduced arterial oxygen (O_2_) tension and/or increased pulmonary ventilation in HH. We hypothesized that HH would reduce cBRS and that this effect would be counteracted by acute restoration of the inspiratory partial pressure of O_2_ ($\text{P}{\text{I}}_{{\text{O}}_{2}}$) and/or voluntary attenuation of pulmonary ventilation. Twelve healthy women (24.0 ± 4.2 yr) were studied before (*day 0*) and twice during a sojourn in a hypobaric chamber (∼8 h, *day 1*; 4 days, *day 4*) where barometric pressure corresponded to ∼3,500-m altitude. Minute ventilation (V̇e; pneumotachometer), heart rate (electrocardiogram), and arterial pressure (finger volume clamp method) were recorded. cBRS was calculated using transfer function analysis between systolic pressure and RR interval. Assessments were made during *1*) spontaneous breathing and (in HH only), *2*) controlled breathing (reducing V̇e by ∼1 to 2 L/min), and *3*) breathing a hyperoxic gas mixture that normalized $\text{P}{\text{I}}_{{\text{O}}_{2}}$. During spontaneous breathing, HH decreased cBRS (12.5 ± 7.1, 8.9 ± 4.4, and 7.4 ± 3.0 ms/mmHg on *days 0*, *1*, and *4*, respectively; *P* = 0.018). The normalization of $\text{P}{\text{I}}_{{\text{O}}_{2}}$ increased cBRS (10.6 ± 3.3 and 10.7 ± 6.1 ms/mmHg on *days 1* and *4*) in HH compared with values observed during spontaneous breathing (*P* < 0.001), whereas controlled breathing had no effect on cBRS (*P* = 0.708). These findings indicate that ongoing arterial chemoreflex activation by the reduced arterial O_2_ tension, independently of the hypoxic ventilatory response, reduces cBRS in young women exposed to extended HH.

**NEW & NOTEWORTHY** We examined the effects of prolonged hypobaric hypoxia (corresponding to ∼3,500-m altitude) on cardiac baroreflex sensitivity (cBRS) in young women and investigated underlying mechanisms. We found that cBRS was reduced in hypoxia and that this reduction was attenuated by acute restoration of inspiratory oxygen partial pressure but not by volitional restraint of pulmonary ventilation. These findings help to elucidate the role of arterial chemoreflex mechanisms in the control of cBRS during hypobaric hypoxia in young women.

## INTRODUCTION

Blood pressure (BP) homeostasis is challenged during hypoxic exposure (1, 2). Among its diverse effects, hypoxia evokes smooth muscle relaxation via endothelium-dependent pathways (3), which tends to reduce peripheral vascular resistance and BP. The resulting unloading of the arterial baroreflex interacts with the input from arterial chemoreceptors and other afferent populations (e.g., cardiac and pulmonary baroreceptors) (4, 5). This influences baroreflex sensitivity and cardiovascular autonomic activity (6, 7), such that sympathoexcitation and cardiac vagal withdrawal maintain or even increase BP (4, 8, 9). The influence of hypoxia on cardiac baroreflex sensitivity (cBRS) has been investigated using a variety of approaches (Supplemental Table S1; all Supplemental material is available at https://doi.org/10.17608/k6.auckland.20514237.v1), and it was generally observed that hypoxia reduces cBRS when quantified in terms of the control of RR interval (reciprocal of heart rate; HR) (7, 10–13). What facilitates this reduction in cBRS is unclear, but ongoing chemoreflex activation by the reduction in arterial oxygen (O_2_) tension that persists even after acclimatization to hypoxia may be involved. Alternatively, the increased pulmonary ventilation in hypoxia could reduce cBRS by virtue of the concomitant lowering of arterial carbon dioxide (CO_2_) tension (14) but also via CO_2_-independent mechanisms (15).

Accordingly, the aim of this study was to assess the effect of extended hypoxia on cBRS and whether this effect is related to reduced arterial O_2_ tension, and/or increased pulmonary ventilation in hypoxia. cBRS was assessed before and again after ∼8 h and 4 days of hypobaric hypoxia (HH; corresponding to ∼3,500-m altitude) exposure. Further assessments were made (in HH only) during the acute restoration of inspiratory O_2_ partial pressure ($\text{P}{\text{I}}_{{\text{O}}_{2}}$), and voluntary attenuation of pulmonary ventilation. It was hypothesized that HH decreases cBRS and that this effect is counteracted by the restoration of $\text{P}{\text{I}}_{{\text{O}}_{2}}$ and the attenuation of pulmonary ventilation. We studied young women because, despite sex differences in cardiovascular, autonomic, and respiratory parameters being reported at sea level and during hypoxia (16–18), neither the effect of prolonged HH on cBRS nor the potential underlying mechanisms have been investigated in women.

## METHODS

### Ethical Approval

The study was approved by the Ethics Committee of the Bolzano Hospital, Italy (No. 70-2019) and conducted according to the Declaration of Helsinki, except for registration in a clinical trials database. Study volunteers provided written informed consent for participation after being provided with a detailed explanation of the study procedures. The data were collected as part of a larger project investigating altitude acclimatization in women; however, the hypotheses are unique, and the experiments used to test them not reported elsewhere.

### Participant Characteristics

Twelve healthy women (24.0 ± 4.2 yr, 59.6 ± 7.4 kg, 168 ± 8 cm) with no history of high altitude-related illness were enrolled. No participant reported being an abuser of alcohol or a user of either tobacco, nicotine-containing products, or recreational drugs. None of the participants was using over-the-counter or prescription medications aside from combined oral (*n* = 11) or progestin intrauterine (*n* = 1) contraception. Experiments were conducted during the active phase of pill consumption and in the absence of menstruation for the progestin user.

### Protocol

Participants were tested on three separate occasions, once before and twice during a 4-day sojourn in a hypobaric chamber (263 m; terraXcube, Eurac Research, Bolzano, Italy), where barometric pressure was reduced to 493.5 mmHg (corresponding to ∼3,500-m altitude). Participants reported to our laboratory the afternoon preceding the sojourn, where the first experimental session was conducted (*day 0*, time point 16:30–18:30). They then spent the night at our facilities and entered the chamber the next morning at 06:00, where they were decompressed at a rate simulating an ascent of 2 m/s. On the 1st and 4th days in the chamber (*days 1* and *4*, time point 14:00–16:00), further experimental sessions were performed. Throughout the sojourn, participants followed the same standardized diet (1,970 kcal/day; 48.5% carbohydrates, 36.9% fat and 14.6% protein, 94.5 and 58.8 mmol/day, Na^+^ and K^+^, 2 L/day water) as during the 4 days preceding chamber entry. They furthermore wore a pedometer and were instructed to reproduce their habitual daily step count as measured before chamber entry. The chamber was maintained at 22°C with a relative humidity of 30%.

Each experimental session commenced with participants reclining in a semirecumbent position while being instrumented for cardiorespiratory monitoring. The experimental session on *day 0* involved *1*) 5 min of spontaneous breathing and *2*) 5 min of controlled breathing, where subjects were instructed to pace their respiratory frequency (R*f*, using a metronome) and tidal volume (V_T,_ measured breath by breath and visible to the subject) to the averaged values from the preceding spontaneous breathing (on *day 0*, this controlled breathing was performed for training purposes only and data are not reported). The experimental sessions in hypoxia (*days 1* and *4*) involved *1*) 5 min of spontaneous breathing; *2*) 5 min of controlled breathing with R*f* paced and V_T_ guided to values measured during spontaneous breathing on *day 0*, followed by a 5-min washout (return to spontaneous breathing); *3*) 5 min of spontaneous breathing a hyperoxic gas mixture that normalized $\text{P}{\text{I}}_{{\text{O}}_{2}}$. The required O_2_ fraction (32%) was calculated as Fo_2_ = $\text{P}{\text{I}}_{{\text{O}}_{2}}$__263m_/(P_B_, 47 mmHg), where P_B_ was the barometric pressure in the chamber, $\text{P}{\text{I}}_{{\text{O}}_{2}}$__263m_, the $\text{P}{\text{I}}_{{\text{O}}_{2}}$ typically encountered at 263-m altitude (145 mmHg), and 47 mmHg, the water vapor pressure in saturated air at 37°C.

### Measurements

Participants were instrumented for the measurement of peripheral oxygen saturation ($\text{S}{\text{p}}_{{\text{O}}_{2}}$; ML320 Oximeter Pod, ADInstruments, Sydney, Australia), HR using a lead II electrocardiogram (Bio Amp, ADInstruments), and continuous arterial blood pressure (BP) using finger photoplethysmography (NOVA, Finapres Medical System, Enschede, The Netherlands). An oronasal mask (Cosmed, Rome, Italy) was worn and connected to a spirometer (Spirometer, ADInstruments) for the measurement of R*f*, V_T_, and minute ventilation (V̇e). For technical reasons, ventilation was not monitored during the hyperoxic gas breathing.

### Data Analysis

Raw signals underwent analog-to-digital conversion at 1 kHz (Powerlab and LabChart v8; ADInstruments) and were stored for offline analysis using Ensemble V1 (Elucimed, Wellington, New Zealand). Systolic (SBP) and diastolic (DBP) BP were obtained from the arterial BP waveform. Mean arterial pressure (MAP) was obtained by the integration of the arterial BP waveform over the entire cardiac cycle.

Spontaneous cBRS was calculated using transfer function analysis between SBP and RR interval (19). Beat-to-beat SBP and RR intervals were identified and time aligned before undergoing spline interpolation and downsampling to 4 Hz to provide equidistant data sets for spectral and transfer function analysis using the Welch algorithm. Briefly, each time series was subdivided into five segments, each overlapping by 50%, before being linearly detrended and passed through a Hanning window and undergoing fast-Fourier transformation analysis. The cross-spectrum between SBP and RR interval was determined and divided by the SBP autospectrum. Mean values of SBP and RR interval spectral power, along with transfer function gain, phase, and coherence, were calculated in the very-low-frequency (<0.04 Hz), low-frequency (0.04–0.15 Hz), and high-frequency (0.15–0.40 Hz) ranges. Low-frequency transfer function gain was used as an index of cBRS (cBRS gain) (20).

HR variability was assessed following the guidelines provided by the Task Force of the European Society of Cardiology and the North American Society of Pacing and Electrophysiology (21). Time-domain HR variability assessment comprised of the square root of the mean of the sum of successive differences in RR interval (RMSSD) and proportion of successive R-R intervals that varied by >50 ms (pNN50%). Frequency-domain analysis was undertaken using fast-Fourier transformation, and the power spectra were quantified by the following frequency bands: very-low-frequency power (<0.04 Hz), low-frequency power (0.04–0.15 Hz), and high-frequency power (0.15–0.40 Hz). Normalized units were calculated by dividing each spectral band by the total power minus the very low-frequency power and multiplied by 100.

Of the 12 participants recruited, a complete data set is available for *n* = 11, as one participant developed frequent ectopic heartbeats in HH and therefore was not suitable for cBRS and HR variability analysis. Because of a technical problem, $\text{S}{\text{p}}_{{\text{O}}_{2}}$ was not measured on *day 0* in two participants, and these missing values were imputed for statistical purposes.

### Statistical Analysis

Normality was assessed using the Shapiro–Wilk test. Nonnormally distributed data underwent logarithmic transformation before further statistical analysis. Primary statistical analyses were performed with repeated-measures analysis of variance (ANOVA) with post hoc analysis undertaken using a Student–Neuman–Keuls test (SigmaPlot version 14.0, Systat Software, San Jose, CA). *P* < 0.05 was considered significant. Values are presented as means ± SD for normally distributed data and medians (interquartile range) for nonnormally distributed data.

## RESULTS

### Effect of HH on cBRS

**Figure 1.**
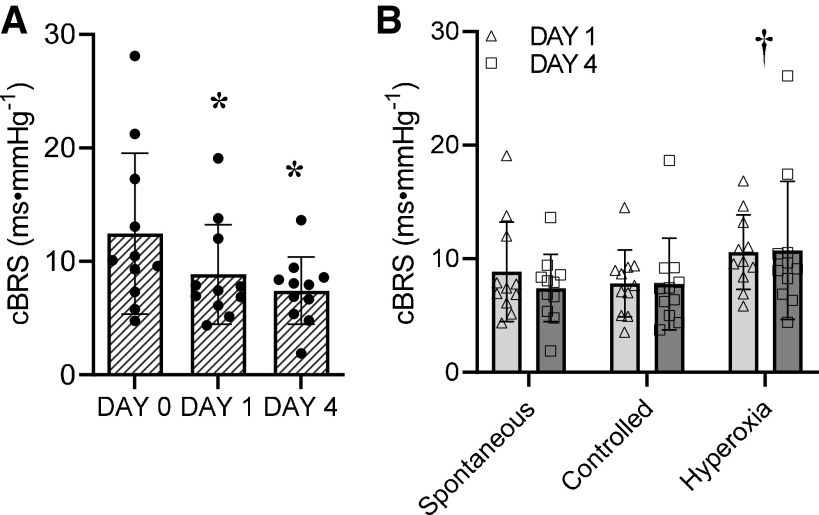
Cardiac baroreflex sensitivity (cBRS) during spontaneous free breathing in normoxia (*day 0*) and hypobaric hypoxia (*days 1* and *4*; *A*) vs. acute normalization of the partial pressure of inspired oxygen and vs. controlled breathing to restrain the hypoxic ventilatory response (*B*). Vertical bars show means and SD. Symbols represent individual values (*n* = 11). *A*: one-way repeated-measures ANOVA. *P < 0.05 vs. *day 0*. *B*: two-way repeated-measures ANOVA. †*P* < 0.05 vs. spontaneous and controlled.

As expected, $\text{S}{\text{p}}_{{\text{O}}_{2}}$ was reduced on *days 1* and *4* of HH (*P* < 0.001), whereas V̇e was increased (*P* < 0.001), principally because of an increase in R*f* (*P* = 0.032; Table 1). SBP, DBP, and MAP were unchanged (*P* > 0.05), whereas HR was elevated (*P* < 0.001) in HH. Conversely, RR interval (*P* < 0.001) and cBRS were reduced in HH (*P* = 0.018; Fig. 1). Absolute LF (*P* = 0.003) and HF power (*P* < 0.001), along with pNN50 (*P* < 0.001) and RMSSD (*P* < 0.001), were also reduced in HH (Table 1).

**Table 1.** Cardiorespiratory variables, cBRS, and HR variability during spontaneous free breathing in normoxia (day 0) and HH (days 1 and 4)

	*Day 0*	*Day 1*	*Day 4*	*P* Value
$\text{S}{\text{p}}_{{\text{O}}_{2}}$, %	97 ± 1	86 ± 4*	88 ± 3*	**<0.001**
V̇e, L/min	11 ± 1	13 ± 1*	14 ± 1*	**<0.001**
R*f*, breaths/min	14 ± 4	16 ± 5	18 ± 3*	**0.032**
V_T_, L	0.82 ± 0.16	0.86 ± 0.15	0.81 ± 0.10	0.488
SBP, mmHg	126 ± 14	123 ± 16	125 ± 8	0.752
DBP, mmHg	78 [69–86]	80 [69–85]	80 [74–84]	0.911
MAP, mmHg	102 [89–109]	99 [87–107]	100 [97–102]	0.839
HR, beats/min	69 ± 6	86 ± 12*	80 ± 7*	**<0.001**
RR interval, s	0.89 [0.84–0.94]	0.74 [0.69–0.76]*	0.75 [0.70–0.83]*	**<0.001**
cBRS LF phase, rad	−0.97 ± 0.28	−1.10 ± 0.24	−1.06 ± 0.19	0.203
cBRS LF coherence	0.62 ± 0.12	0.63 ± 0.16	0.55 ± 0.18	0.095
VLF power, ms^2^	147 [109–219]	76 [43–104]	65 [50–174]	0.076
LF power, ms^2^	471 [216–926]	303 [214–363]*	272 [147–401]*	**0.003**
HF power, ms^2^	1,944 [980–2,825]	554 [206–660]*	304 [208–1,108]*	**<0.001**
LF power, %	31 ± 21	44 ± 25	38 ± 18	0.198
HF power, %	67 ± 22	51 ± 26	57 ± 18	0.148
LF/HF, AU	0.27 [0.21–0.81]	0.60 [0.40–2.20]	0.68 [0.37–1.42]	0.152
pNN50, %	52 ± 27	24 ± 21*	30 ± 27*	**<0.001**
RMSSD	85 ± 53	47 ± 41*	50 ± 31*	**<0.001**

Values are means ± SD for normally distributed data and medians [interquartile ranges] for non-normally distributed data. HR, heart rate; HH, hypobaric hypoxia; $\text{S}{\text{p}}_{{\text{O}}_{2}}$, arterial oxygen saturation; V̇e, minute ventilation; R*f*, respiratory frequency; V_T_, tidal volume; SBP, systolic blood pressure; DBP, diastolic blood pressure; MAP, mean arterial pressure; cBRS, cardiac baroreflex sensitivity; LF, low frequency; VLF, very-low frequency; HF, high frequency; AU, arbitrary units; pNN50, proportion of successive R–R intervals that varied by >50 ms; RMSSD, square root of the mean of the sum of successive differences in RR interval. Between-day differences were examined using one-way ANOVA with repeated measures. Significant differences observed during post hoc analyses (Student–Newman–Keuls method) are shown as *P < 0.05 vs. *day 0*. Boldface indicates signifcant values.

### cBRS during Acute Normalization of $\text{P}{\text{I}}_{{\text{O}}_{2}}$

Normalization of $\text{P}{\text{I}}_{{\text{O}}_{2}}$ restored $\text{S}{\text{p}}_{{\text{O}}_{2}}$ (*P* < 0.001), increased cBRS (*P* < 0.001), and reduced HR (*P* = 0.005; Table 2 and Fig. 1). Moreover, normalization of $\text{P}{\text{I}}_{{\text{O}}_{2}}$ increased LF power (*P* = 0.043) but did not affect SBP, DBP, MAP, or any other HR variability index.

**Table 2.** Cardiorespiratory, cBRS, and HR variability parameters during spontaneous breathing and with acute normalization of $\text{P}{\text{I}}_{{\text{O}}_{2}}$ (hyperoxia) during HH

	*Day 1*		*Day 4*		ANOVA *P* Value		
	Spontaneous	Hyperoxia	Spontaneous	Hyperoxia	Trial	Day	Inter
$\text{S}{\text{p}}_{{\text{O}}_{2}}$, %	86 ± 4	97 ± 2	88 ± 3	97 ± 1	**<0.001**	**0.032**	0.112
SBP, mmHg	124 [109–136]	122 [116–137]	127 [123–129]	122 [117–125]	0.720	0.893	0.284
DBP, mmHg	80 [69–85]	79 [72–86]	80 [74–84]	77 [72–83]	0.841	0.935	0.389
MAP, mmHg	99 [87–107]	99 [92–106]	100 [97–102]	100 [92–101]	0.799	0.851	0.267
HR, beats/min	82 [79–87]	79 [72–80]	80 [73–87]	78 [73–79]	**0.005**	0.132	0.110
RR interval, s	0.74 [0.69–0.76]	0.76 [0.75–0.84]	0.75 [0.70–0.83]	0.77 [0.76–0.83]	**0.004**	0.148	0.117
cBRS LF phase, rad	−1.13 [−1.27 to −0.99]	−1.03 [−1.19 to −0.85]	−1.00 [−1.19 to −0.93]	−1.22 [−1.25 to −1.02]	0.486	0.216	0.190
cBRS LF coherence	0.66 [0.56–0.72]	0.62 [0.56–0.71]	0.59 [0.52–0.68]	0.59 [0.54–0.68]	0.394	0.062	0.303
VLF power, ms^2^	145 ± 237	160 ± 160	151 ± 194	109 ± 141	0.780	0.613	0.362
LF power, ms^2^	303 [214–363]	429 [316–654]	272 [147–401]	370 [168–552]	**0.043**	0.217	0.953
HF power, ms^2^	554 [206–660]	390 [190–1,092]	304 [208–1,108]	487 [254–961]	0.378	0.587	0.728
LF power, %	44 ± 25	49 ± 19	38 ± 18	43 ± 17	0.287	0.301	0.946
HF power, %	51 ± 26	45 ± 19	57 ± 18	51 ± 16	0.191	0.302	0.980
LF/HF, AU	0.60 [0.40–2.20]	1.49 [0.66–1.78]	0.68 [0.37–1.42]	0.58 [0.46–1.39]	0.290	0.277	0.811
pNN50, %	18 [11–36]	25 [16–38]	17 [12–44]	22 [20–28]	0.202	0.940	0.443
RMSSD	38 [31–51]	50 [38–53]	37 [32–57]	41 [39–60]	0.113	0.332	0.207

Values are means ± SD for normally distributed variables and medians [interquartile ranges] for non-normally distributed data. HR, heart rate; $\text{P}{\text{I}}_{{\text{O}}_{2}}$, inspiratory partial pressure of oxygen; HH, hypobaric hypoxia; $\text{S}{\text{p}}_{{\text{O}}_{2}}$, arterial oxygen saturation; SBP, systolic blood pressure; DBP, diastolic blood pressure; MAP, mean arterial pressure; cBRS, cardiac baroreflex sensitivity; LF, low frequency; VLF, very-low frequency; HF, high frequency; AU, arbitrary units; pNN50, proportion of successive R–R intervals that varied by >50 ms; RMSSD, square root of the mean of the sum of successive differences in RR interval. The main effects of trial (spontaneous, hyperoxia), day (*day 1*, *day 2*), and their interaction (Inter) were examined using two-way ANOVA with repeated measures. Boldface indicates signifcant values.

### cBRS during Attenuation of Pulmonary Ventilation

Even though subjects had difficulties reducing R*f* and/or V_T_ to the levels observed on *day 0*, the controlled breathing still reduced V̇e by 1 to 2 L/min compared with spontaneous breathing (*P* = 0.016; Table 3). This reduction in V̇e, however, did not affect cBRS (*P* = 0.708; Fig. 1). However, SBP was elevated (*P* = 0.005) and LF power decreased (*P* = 0.011) by the controlled breathing (Table 3).

**Table 3.** Cardiorespiratory, cBRS, and HR variability parameters in HH with spontaneous and controlled breathing

	*Day 1*		*Day 4*		ANOVA *P* Value		
	Spontaneous	Controlled	Spontaneous	Controlled	Trial	Day	Inter
$\text{S}{\text{p}}_{{\text{O}}_{2}}$, %	87 [82–88]	85 [83–87]	88 [87–88]	86 [86–88]	0.244	**0.018**	0.881
V̇e, L/min	13 [13–14]	12 [12–13]	14 [13–14]	12 [12–13]	**0.016**	0.110	0.236
R*f*, breaths/min	16 ± 5	15 ± 3	18 ± 3	15 ± 3	0.160	0.295	0.344
V_T_, L	0.86 ± 0.15	0.85 ± 0.13	0.81 ± 0.09	0.86 ± 0.11	0.585	0.429	0.255
SBP, mmHg	124 [109–136]	133 [120–143]	127 [123–129]	137 [128–143]	**0.005**	0.301	0.262
DBP, mmHg	80 [69–85]	79 [73–92]	80 [74–84]	83 [80–91]	0.061	0.508	0.310
MAP, mmHg	97 ± 12	103 ± 17	99 ± 6	108 ± 9	**0.019**	0.348	0.291
HR, beats/min	86 ± 12	88 ± 8	80 ± 7	83 ± 9	0.349	**0.025**	0.767
RR interval, s	0.71 ± 0.09	0.69 ± 0.06	0.76 ± 0.07	0.74 ± 0.08	0.196	**0.014**	0.987
cBRS LF phase, rad	−1.13 [−1.27 to −0.99]	−1.04 [−1.10 to −0.96]	−1.00 [−1.19 to −0.93]	−1.08 [−1.19 to −0.91]	0.469	0.795	0.690
cBRS LF coherence	0.63 ± 0.16	0.62 ± 0.15	0.55 ± 0.17	0.55 ± 0.13	0.922	**0.046**	0.761
VLF power, ms^2^	145 ± 237	72 ± 81	151 ± 194	63 ± 55	0.110	0.963	0.639
LF power, ms^2^	390 ± 321	302 ± 304	316 ± 246	232 ± 228	**0.011**	0.128	0.922
HF power, ms^2^	554 [206–660]	423 [159–811]	304 [208–1,108]	617 [285–1,049]	0.564	0.145	0.236
LF power, %	44 ± 25	40 ± 25	38 ± 18	25 ± 13	**0.031**	0.067	0.126
HF power, %	51 ± 26	57 ± 24	57 ± 18	71 ± 13	**0.049**	0.071	0.224
LF/HF, AU	0.60 [0.40–2.20]	0.47 [0.24–1.66]	0.68 [0.37–1.42]	0.24 [0.20–0.45]	0.054	0.062	0.312
pNN50, %	24 ± 21	18 ± 21	30 ± 27	26 ± 23	0.069	**0.048**	0.680
RMSSD	38 [31–51]	34 [25–49]	37 [32–57]	38 [28–49]	0.362	0.446	0.980

Values are means ± SD for normally distributed variables and medians [interquartile range] for non-normally distributed data. HR, heart rate; HH, hypobaric hypoxia; $\text{S}{\text{p}}_{{\text{O}}_{2}}$, arterial oxygen saturation; V̇e, minute ventilation; R*f*, respiratory frequency; V_T_, tidal volume; SBP, systolic blood pressure; DBP, diastolic blood pressure; MAP, mean arterial pressure; cBRS, cardiac baroreflex sensitivity; LF, low frequency; VLF, very-low frequency; HF, high frequency; AU, arbitrary units; pNN50, proportion of successive R–R intervals that varied by >50 ms; RMSSD, square root of the mean of the sum of successive differences in RR interval. The main effects of trial (spontaneous, controlled), day (*day 0*, *day 1*, *day 2*), and their interaction (Inter) were examined using two-way ANOVA with repeated measures. Boldface indicates signifcant values.

## DISCUSSION

We investigated the effect of moderate HH on cBRS in young women and whether this effect is related to reduced arterial O_2_ tension and/or increased pulmonary ventilation in HH. As anticipated, cBRS was reduced after both 8 h and 4 days in HH. In line with our hypotheses, acute normalization of $\text{P}{\text{I}}_{{\text{O}}_{2}}$ increased cBRS, whereas, contrary to our hypotheses, cBRS was not affected by volitional attenuation of pulmonary ventilation in HH. Collectively, these findings support that arterial chemoreflex activation by the reduced arterial O_2_ tension, independently of the hypoxic ventilatory response, reduces cBRS in young women exposed to HH.

This study is the first to demonstrate that initial reductions in cBRS with acute hypoxia in women persist throughout extended (4 days) HH, in accordance with studies in men and combined groups of men and women. This was important to establish because sex differences in cBRS (16), HR variability (17), chemoreflex sensitivity (22, 23), and neurovascular regulation (24) at sea level and in hypoxia indicate that findings from investigations of cardiorespiratory regulation in men cannot necessarily be extrapolated to women.

The second novel aspect of this study was the exploration of the mechanism through which HH attenuates cBRS in women. Activation of the arterial chemoreflex by the reduced arterial O_2_ tension seemed a likely candidate, and this is supported by the finding that acute restoration of $\text{P}{\text{I}}_{{\text{O}}_{2}}$ increased cBRS on both *day 1* and *day 4* in HH. In contrast to arterial O_2_ content, which normalizes within a few days of hypoxic exposure, arterial O_2_ tension increases only modestly with acclimatization, presumably explaining why the hypoxia-induced cBRS reduction persists during prolonged hypoxic exposure. Yazdani et al. (25) even observed that the initial HH-induced reduction in cBRS became more pronounced after 16 days of exposure, potentially as sensitization and long-term facilitation augmented chemoreflex activation by the reduced arterial O_2_ tension. On the other hand, the reductions in cBRS were reversed by hyperoxic gas breathing after 2–4 h but not on the 16th day (25) of HH exposure, perhaps indicating that non-chemoreflex mechanisms are also involved at this latter time point. Taken together, these observations indicate that the duration of hypoxic exposure can affect the mechanisms underlying the reductions in cBRS.

We also speculated that the increase in V̇e that results from the hypoxic exposure and subsequent ventilatory acclimatization could contribute to the reduction in cBRS in HH, either through the concomitant lowering in arterial CO_2_ tension (14) or via CO_2_-independent mechanisms (15). For example, Bourdillon et al. (14) reported that hyperventilation sufficient to lower the partial pressure of end-tidal CO_2_ (a proxy for arterial CO_2_ tension) to ∼20 mmHg markedly reduced cBRS. However, Van De Borne et al. (15) observed that cBRS was also reduced during isocapnic hyperventilation. Potential CO_2_-independent mechanisms whereby cBRS is reduced include pulmonary stretch receptor activation. In the present study, we observed that controlled breathing to restrain the hyperpnea, which accompanies prolonged hypoxic exposure failed to increase cBRS. It must be emphasized that the reduction in V̇e induced by the controlled breathing was modest and that we cannot exclude that a more pronounced reduction would have had an effect. Nevertheless, suppression of ventilation induces discomfort, and this effect is likely augmented in hypoxia where the ventilatory sensitivity to reductions in arterial O_2_ and increases in arterial CO_2_ is augmented. As such, a more pronounced voluntary attenuation of ventilation in HH would have presumably required an extended training period.

Alterations in cardiac autonomic activity, specifically reductions in vagal activity (26, 27) but possibly also increases in sympathetic activity (28), are likely associated with the hypoxia-mediated reduction in cBRS. It is well established that hypoxia induces an increase in sympathetic nerve activity which persists throughout prolonged exposure (4, 5, 8), but intriguingly this sympathoexcitation is only modestly affected by acute inhibition of carotid chemoreflex activation with either hyperoxia (4) or low-dose dopamine infusion (8) and even persists for several days after return to normoxia (4). The effects of extended hypoxia on cardiovagal activity have been less studied. Nevertheless, we have recently used autonomic blockade to isolate vagal control of HR and demonstrated that vagal withdrawal persists throughout 2 wk of HH exposure (9). Also in the current study, the analysis of HR variability-derived indices of cardiovagal control (i.e., HF power, pNN50, RMSSD) supports reduced vagal activity on both *day 1* and *day 4* in HH. That these indices of cardiovagal control were not affected by the acute normalization of $\text{P}{\text{I}}_{{\text{O}}_{2}}$ supports that similar to hypoxia-induced sympathoexcitation, hypoxia-induced vagal withdrawal is not acutely reversed when the hypoxic stimulus ceases.

There are methodological aspects to consider. cBRS was calculated using transfer function analysis between SBP and RR interval (19), an approach that, similar to the sequence technique, provides an assessment of cBRS at the operating point of the full arterial baroreflex stimulus-response relationship (26). The hyperoxic gas mixture presumably not only restored arterial O_2_ tension in HH but may have also attenuated V̇e and thus increased arterial CO_2_ tension. However, since the reduction in V̇e and/or an increase in arterial CO_2_ tension induced by controlled breathing had no effect on cBRS, it is unlikely that attenuation of V̇e contributed to the reduction in cBRS induced by the hyperoxic gas mixture. Participants all used hormonal contraception and were tested during the active phase of pill consumption to limit the potentially confounding effect of variations in ovarian hormones concentration in this longitudinal study and to provide data representative of the significant proportion of women using hormonal contraception. It remains to be investigated whether/how the effect of HH on cBRS interacts with the menstrual cycle. A final experimental consideration is the use of laboratory-based setting using a hypobaric chamber rather than a high-altitude sojourn. Although both have relative strengths and limitations (29), the advantage of using a hypobaric chamber is that the hypoxic stimulus can be selectively applied without potential confounding (e.g., by cold, humidity, solar radiation, physical activity, sleep, and diet).

In summary, we found that cBRS was reduced by prolonged HH in young women. This reduction was attenuated by acute restoration of inspiratory O_2_ partial pressure but not by volitional restraint of pulmonary ventilation. This suggests that hyperpnea per se does not explain the reduction in cBRS in HH and points to a predominant role for the arterial chemoreflex in reducing cBRS.

## SUPPLEMENTAL DATA

10.17608/k6.auckland.20514237.v1 Supplemental Table S1: https://doi.org/10.17608/k6.auckland.20514237.v1.

## GRANTS

J.P.F. is supported by Auckland Medical Research Foundation Grant 1119008 and Health Research Council of New Zealand Grant 19/687. C.S. is supported by the Swiss National Center of Competence in Research (NCCR) Kidney Control of Homeostasis Grant N-403-03-51 (Kidney.ch) and the Herbert N. Hultgren Grant (Wilderness Medical Society, USA).

## DISCLOSURES

No conflicts of interest, financial or otherwise, are declared by the authors.

## AUTHOR CONTRIBUTIONS

J.P.F., J.R., and C.S. conceived and designed research; J.R., R.T., A.W., G.R., H.G., and C.S. performed experiments; J.P.F. analyzed data; J.P.F., J.R., and C.S. interpreted results of experiments; J.P.F. prepared figures; J.P.F. drafted manuscript; J.P.F., J.R., R.T., H.G., and C.S. edited and revised manuscript; J.P.F., J.R., R.T., A.W., G.R., H.G., and C.S. approved final version of manuscript.
